# Whole-Body Vibration to Improve Physical Function Parameters in Nursing Home Residents Older Than 80 Years: A Systematic Review With Meta-Analysis

**DOI:** 10.1093/ptj/pzae025

**Published:** 2024-02-29

**Authors:** Borja Sañudo, Gonzalo Reverte-Pagola, Adérito Seixas, Tahir Masud

**Affiliations:** Departamento de Educación Física y Deporte, Universidad de Sevilla, Seville, Spain; Departamento de Educación Física y Deporte, Universidad de Sevilla, Seville, Spain; Escola Superior de Saúde Fernando Pessoa, Fisioterapia, Porto, Portugal; Nottingham University Hospitals NHS Trust, Department of Geriatric Medicine, Nottingham, United Kingdom

**Keywords:** Balance, Institutionalized, Mobility, Physical Performance, Whole-Body Vibration

## Abstract

**Objective:**

Loss of functional independence is more likely in older adults who reside in an institution as a consequence of a decline in muscle mass and a loss of force production capacity. The aim of this review was to assess the effect of whole-body vibration (WBV) interventions on the strength, balance, and mobility of nursing home residents older than 80 years.

**Methods:**

An electronic search in MEDLINE, Scopus, and CINAHL databases was conducted. Randomized controlled trials that involved nursing home residents older than 80 years, that investigated WBV interventions compared with nonintervention, usual care, or placebo, and that measured physical function outcomes including strength, balance, gait, and mobility were included. Risk of bias was assessed by 2 reviewers using the Physiotherapy Evidence Database scale. The standardized mean differences (SMD) between the experimental and control groups were calculated with a random-effects model for each outcome, and subgroup analysis was conducted for different outcomes.

**Results:**

In total, 2864 articles were identified; of these, 14 randomized controlled trials met the inclusion criteria. The meta-analysis revealed that WBV significantly increased the lower limb muscle strength (SMD = 0.59; 95% CI = 0.16 to 1.03), mobility (SMD = 0.45; 95% CI = 0.10 to 0.81), gait score (SMD = 0.26; 95% CI = 0.01 to 0.51), balance (SMD = 0.41; 95% CI = 0.01 to 0.81), and physical performance (SMD = 1.33; 95% CI = 0.33 to 2.33).

**Conclusion:**

WBV may be an effective intervention to improve the strength, balance, mobility, walking ability, and physical performance of older nursing home residents.

**Impact:**

WBV presents a safe, accessible alternative for improving health in this vulnerable population, warranting further research and integration into health care practices.

## Introduction

Societies are progressively aging; in particular, there is a growing population older than 80 years.^1^ This aging population presents unique challenges in terms of health and physical capabilities. Although there is notable heterogeneity in health and function among older adults,^2^ these projections of life expectancy are usually associated with physical impairments and functional limitations.^3^ The resulting functional decline can affect the ability to perform activities of daily living in this population group.^4^ Consequently, the loss of functional independence is an important risk factor for institutionalization.^3^ Sarcopenia and frailty are also increasingly prevalent in this population and are associated with greater dependency and mortality.^5^ The former is characterized by loss of muscle mass and strength and decreased physical performance,^6^ and the latter, is a multidimensional geriatric syndrome that has a negative impact in several dimensions (ie, social, cognitive, and physical).^5^

Larsson et al^7^ recently described most of the factors that contribute to the age-related loss of muscle function. Aging is accompanied by a loss of force production capacity and a decline in muscle mass, which can become more pronounced during periods of inactivity, often experienced by older adults who reside in an institution.^8^ Maintaining an active lifestyle late in life is considered to be one of the main strategies that can overcome these limitations and has a considerable impact on daily life activities.^1^ Physical exercise is considered a cornerstone in preserving physical function in older adults^9^ and numerous meta-analyses have suggested improvements in overall physical performance (including gait speed, mobility, or balance) and activities of daily living in frail older people.^10–12^ As the degree of benefit depends on the type of exercise performed, a recent meta-analysis showed the effectiveness of resistance training for improving muscle strength and power as well as functional outcomes in very old (older than 87 years) people.^13^ However, although evidence supports the benefits of multicomponent exercise programs, such as combinations of walking, balance, and resistance exercises, for improving functional abilities in the oldest old population, it is important to acknowledge that some individuals may face challenges in adhering to these programs.^14^ As a result, alternative strategies may be required to prevent functional decline in these older individuals. Approaches like blood flow restriction,^15^ neuromuscular electrical stimulation,^16^ and whole-body vibration (WBV)^17^ have emerged as potential strategies for older individuals.

WBV stands out as a promising approach, especially when traditional exercise paradigms may not be feasible. This form of exercise elicits involuntary muscle contractions through the activation of stretch reflexes,^18^ thereby making it useful during disuse situations. Several systematic reviews have reported promising results with the use of WBV in improving muscle strength, walking ability, mobility or body balance and in reducing falls in older adults.^19–22^ This intervention was considered to be safe and feasible in very old nursing home residents.^23^ Moreover, recent studies even suggested that the improvements in balance and muscle strength after WBV training in older people who reside in an institution are equivalent to an exercise program without vibration.^24^ However, the literature is not without inconsistencies, and there is no consensus regarding the most suitable WBV protocols for improving functional capacity in older populations, particularly among nursing home residents. Furthermore, the response to WBV might be different when compared with community-dwelling older people.^25^ Given these considerations, we believe there is a compelling need to conduct an updated systematic review to explore the effectiveness of WBV as an intervention for improving physical function among older nursing home residents (80 years old and older). Therefore, our aim in this study was to review systematically the literature, focusing on the identification of WBV interventions that attempt to enhance the strength, balance, mobility, and walking ability of nursing home residents 80 years old and older.

## Material and Methods

### Search Strategy and Selection Criteria

The current systematic review was reported according to Preferred Reporting Items for Systematic Reviews and Meta-Analyses (PRISMA)^26^ and was registered in advance in the International Prospective Register of Systematic Reviews (PROSPERO) as CRD42021279735. Further details (PRISMA checklist) can be found in the Supplementary Materials. A systematic literature search was conducted on MEDLINE (via PubMed), Scopus, and CINAHL, from database inception until July 1, 2023. The search strategies varied according to the different databases searched and used the following search terms: (“nursing home” or “resident” or “long-term care” or “institutional care” or “assisted living” or “institutionalized”) and (“vibration” or “WBV” or “whole-body vibration”) and (“older people” or “elderly” or “older adults” or “aged” or “ageing” or “aging” or “oldest” or “old”). A manual search within the reference lists of retrieved publications was also completed. This systematic review was designed according to the PICOS (patient population, intervention, comparative controls, outcomes) framework, where the population consisted of nursing home residents older than 80 years; the intervention was based on WBV; the comparison was a control group (nonintervention, usual care, or placebo); the outcomes were physical function parameters including strength, balance, gait, and mobility; and the study design had to be a randomized controlled trial. Studies were excluded when the older people did not reside in an institution; the participants were younger than 80 years; the intervention consisted of only the acute effect of a single WBV session or the article types were conference abstracts, letters, or review articles; data were incomplete or articles were duplicate publications; and there was a high risk of bias (Physiotherapy Evidence Database [PEDro] scale score of <4).

### Study Selection and Data Extraction

All articles identified by our search strategy were examined (title and abstract). Screening was performed independently by 2 blinded reviewers (B.S. and G.R.-P.). In cases of disagreement regarding the final list of studies to be included were resolved by consensus by including the third reviewer (A.S.). The main study characteristics (ie, country, participants enrolled, age, relevant outcomes, adherence, and adverse effects) and the intervention parameters (frequency, amplitude, peak acceleration, platform device used, sessions per week, position, protocol, duration, and footwear) were extracted by the reviewers. WBV involves the use of a vibrating platform that induces involuntary muscle contractions through the activation of stretch reflexes. Several parameters play a crucial role in the effectiveness of WBV, including the amplitude (peak displacement in millimeters), frequency (measured in hertz), and the type of platform used. Amplitude represents the extent of vertical displacement during each vibration cycle, which influences the intensity of muscle contractions. Frequency, measured in hertz, denotes the number of vibration cycles per second and determines the stimulation frequency applied to the muscles. The type of platform can vary, including pivotal or oscillating platforms and vertical platforms, each with distinct mechanical characteristics. If the information was incomplete, authors were contacted to supply information or data for inclusion in the meta-analysis.

### Risk of Bias of Individual Studies

The risk of bias was evaluated by 2 reviewers using the PEDro scale score when the score of an article was not shown in the PEDro website. This is considered to be a valid measure of the methodological quality of clinical trials.^27^

### Data Synthesis and Analysis

The software Review Manager 5.4 (Cochrane Training, London, UK) was used to perform a meta-analysis. The physical performance, lower limb muscle strength, gait, balance, and mobility were assessed with diverse instruments, as continuous outcomes. The standardized mean difference (SMD) between the experimental and control group were calculated for each outcome, along with 95% CIs. Subgroup analysis was conducted for a meta-analysis of the different physical outcomes. SMDs were considered statistically significant at the 5% level (*P* ≤ .05) and were classified as small (0.1–0.3), medium (0.3–0.6), or large (>0.6).^28^ When missing, data were requested from the original author by 1 of the researchers. In those studies with 2 intervention groups and a single control group, the sample size of the control group was halved in the statistical analysis to avoid miscalculating the population size.^29^ SMDs were calculated with a random-effects model. Heterogeneity was measured using the *I*^2^ statistics.^30^*I*^2^ values of 25%, 50%, and 75% were considered to represent low, moderate, and considerable heterogeneity.^31^ Studies with a high risk of bias (PEDro scale score of <4) were not considered in the present review.

## Results

### Study Selection

The literature search strategy revealed a total of 2864 possibly eligible articles. After the removal of duplicates, 1382 articles were screened for potential eligibility. After the titles and abstracts were screened, 1279 irrelevant articles were removed. Thus, 103 publications remained for further investigation; 29 of them were excluded because of age, 41 did not involve older adults who resided in an institution, and 1 did not provide enough protocol details. Further, 16 studies were not randomized controlled trials, and 2 did not included physical performance outcomes. Finally, 14 randomized controlled trials^19^^,^^24^^,^^32–43^ were included in this meta-analysis (Supplementary Figure).

### Study Characteristics

The main characteristics of the 14 studies included in this review are summarized in Supplementary Table 1. The 14 studies were conducted in the United States (*n* = 2), Belgium (*n* = 5), Switzerland (*n* = 2), Hong Kong (*n* = 1), Japan (*n* = 1), Finland (*n* = 1), and Spain (*n* = 2) with a total of 672 older adults who resided in an institution (total number analyzed: 566); the mean age of the participants was 83 (SD = 2) years (range = 80–91 years). Most studies included men and women, and the sample sizes in the WBV groups across the studies ranged from 8^39^ to 81^24^ participants. Outcomes included physical function measures assessing knee flexion and extension strength (isometric and dynamic), balance tests, gait speed measurements, Timed “Up & Go” (TUG) Test, Chair Stand Test (CST), and Short Physical Performance Battery (SPPB).

The compliance with the exercise programs was >73% in most studies, and the adherence rate was >90%, with most patients completing >80% of the sessions. No notable side effects were observed in the majority of the studies. Transient minor tingling of the lower limbs, lower limb soreness or pain (knees and lumbar spine), transient itching, headache, and erythema or edema of the legs were common during the beginning of training.

The intervention parameters in the included studies are reported in Supplementary Table 2. The range of the frequency of the mechanical vibrations varied from 3 to 40 Hz, with 8 of the 14 studies starting the exercise program with 30 Hz. The amplitude varied from 0.9 to 8 mm. Consequently, the range of peak acceleration was wide (ie, 1.2–12.4 g). Regarding the type of platforms, both synchronous (vertical)^19^^,^^24^^,^^32–34^^,^^36^^,^^37^^,^^41^^,^^42^ and side-alternating^35^^,^^38–40^^,^^43^ platforms were used in the selected studies. Different positions of the individuals on the base of the platform were adopted. Different variants of the squat exercise were performed in all of the studies, with patients standing on the base of the platform with a slight flexion of their knees (eg, 30 degrees) or even holding onto parallel bars. Nevertheless, in some studies the WBV programs included different exercises, with patients standing on the platform, such as calf raises, single-leg stance, or lateral weight transfers.

Generally, the participants exercised 3 times per week; in 3 studies, there were 2 sessions per week.^37^^,^^40^^,^^41^ The duration of the programs varied from 4 weeks^38^ to 24 weeks.^34^^,^^36^^,^^42^ A description of the protocols used (eg, working time, number of sessions, or rest time) was also provided (Supplementary Tab. 2). The number of sets varied from 1 to 8, with 1 to 12 repetitions; the working time was 15 to 60 seconds in each exercise, and the rest time was 45 to 120 seconds.

### Risk of Bias Within Studies

Eleven of the studies achieved the cutoff score for high quality (≥6), and 3 studies had fair quality (ie, score of 5/10) (Supplementary Tab. 3). Most of the studies failed to conceal allocation or to blind the participants.

### Synthesis of Results

#### Lower Limb Muscle Strength

**Figure 1:**
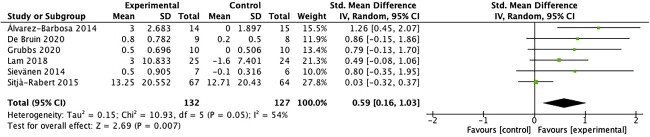
Forest plot of meta-analysis results for lower limb muscle strength estimated with functional tests. IV = inverse variance; Std. = standardized.

Lower limb muscle strength was assessed by both isometric and dynamic tests.^19^^,^^37^^,^^38^^,^^41^^,^^42^ Lower limb muscle strength was also estimated with functional tests (ie, CST or 5 repetition CST) in 6 studies.^19^^,^^24^^,^^32^^,^^37^^,^^40^^,^^43^ Random-effects analysis showed that WBV exercise significantly increased lower limb muscle strength relative to that in controls (SMD = 0.59; 95% CI = 0.16 to 1.03; *P* = .007). Heterogeneity between studies was substantial (*I*^2^ = 54%; *Q* = 10.93; *P* = .05) (Fig. 1).

**Figure 2:**
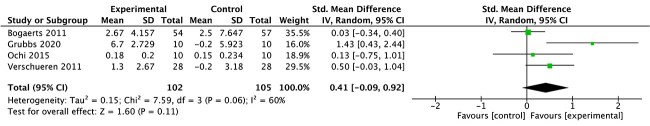
Forest plot of meta-analysis results for lower limb muscle strength estimated with isometric strength tests. IV = inverse variance; Std. = standardized.

By contrast, no significant differences between groups were found in isometric strength^34^^,^^37^^,^^39^^,^^42^ (SMD = 0.41; 95% CI = −0.09 to 0.92; *P* = .11; *I*^2^ = 60%; *Q* = 7.59; *P* = .06) (Fig. 2).

#### Mobility

**Figure 3:**
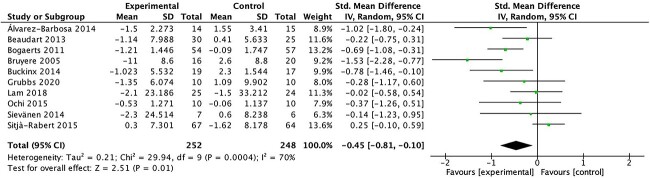
Forest plot of meta-analysis results for mobility estimated with the Timed “Up & Go” Test. IV = inverse variance; Std. = standardized.

Mobility was assessed by most of the included studies. Ten of the 14 included studies assessed mobility with the TUG Test. The meta-analyses showed a significant improvement in mobility in the exercise group (SMD = 0.45; 95% CI = 0.10 to 0.81; *P* = .01). Heterogeneity between studies was substantial (*I*^2^ = 70%; *Q* = 29.94; *P <* .001) (Fig. 3).

#### Walking Speed

**Figure 4:**
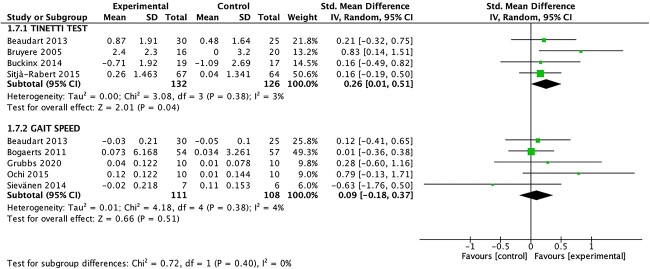
Forest plot for subgroup analysis of gait outcomes. IV = inverse variance; Std. = standardized.

Gait outcomes were assessed in 4 studies using the Tinetti Test,^24^^,^^33–35^ whereas 5 studies^33^^,^^34^^,^^37^^,^^39^^,^^40^ assessed walking speed over 2.44-, 4-, or 10-m walking courses. The meta-analyses (Fig. 4) showed that WBV exercise significantly increased gait score relative to those in controls (SMD = 0.26; 95% CI = 0.01 to 0.51; *P* = .04). However, nonsignificant differences in gait speed (SMD = 0.20; 95% CI = −0.25 to 0.64; *P* = .51) were observed for participants in the WBV training group compared with those in the control group, overall with nonsignificant heterogeneity (*I*^2^ = 4%; *P* = .40).

#### Balance

**Figure 5:**
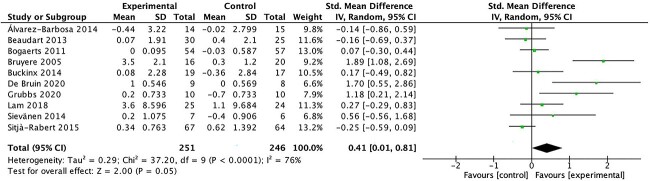
Forest plot of meta-analysis results for balance. IV = inverse variance; Std. = standardized.

Ten studies assessed balance, 4 with the Tinetti Test,^24^^,^^33^^,^^35^^,^^36^^,^^44^ 2 with postural sway,^32^^,^^34^ 3 with a subscale of the SPPB assessing a participant’s ability to stand in 3 different standing positions (semitandem, side by side, and tandem for 10 seconds),^37^^,^^40^^,^^43^ and 1 with the Berg Balance Scale.^19^ The meta-analyses (Fig. 5) showed significant changes in balance (SMD = 0.41; 95% CI = 0.01 to 0.81; *P* = .05) for participants in the WBV training group compared with those in the control group, overall with significant heterogeneity (*I*^2^ = 76%; *P* < .001).

#### Short Physical Performance Battery

**Figure 6:**
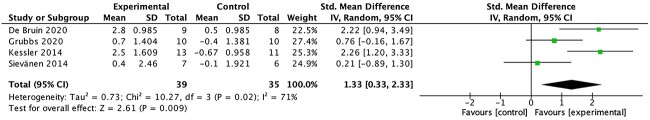
Forest plot of meta-analysis results for physical performance (Short Physical Performance Battery). IV = inverse variance; Std. = standardized.

Four studies assessed the effect of WBV on physical performance (SPPB). Random-effects analysis showed a significant pooled difference between the intervention and control groups (*P* = .009). The SMD was 1.33, and the 95% CI was 0.33 to 2.33, with significant heterogeneity across studies (I^2^ = 71%; *P* = .02) (Fig. 6).

## Discussion

The purpose of this review was to identify and assess the effectiveness of WBV-based interventions in enhancing strength, balance, mobility, walking ability and the physical performance of nursing home residents 80 years old and older. Although a previous meta-analysis showed a significant positive effect of WBV on the TUG Test but not in balance outcomes in older people who resided in an institution,^25^ our meta-analysis revealed that WBV significantly improved lower limb muscle strength, mobility, gait score, balance, and physical performance in people who resided in an institution and were older than 80 years.

The observed improvements in lower limb muscle strength, mobility, gait, balance, and physical performance following WBV exercise interventions hold important clinical implications. For lower limb muscle strength, our meta-analysis revealed a significant SMD of 0.59 (95% CI = 0.16 to 1.03; *P* = .007). Although the heterogeneity across studies was substantial, this increase in lower limb muscle strength suggests that WBV could translate into meaningful changes in functional capacity for older adults. Importantly, although isometric strength did not show significant improvements, the impact of WBV on dynamic lower limb muscle strength appears promising. Mobility, as assessed by the TUG Test, also exhibited significant improvement (SMD = 0.45; 95% CI = 0.10 to 0.81; *P* = .01), indicating that WBV can enhance the ability of older individuals to perform essential daily tasks safely and efficiently. Gait outcomes, although significant in terms of gait score, did not translate to a significant increase in gait speed. However, these findings, together with the significant improvement in balance (SMD = 0.41; 95% CI = 0.01 to 0.81; *P* = .05), suggest that WBV may contribute to fall prevention, enhanced stability, and overall functional independence. Notably, the significant improvement in the SPPB score (SMD = 1.33; 95% CI = 0.33 to 2.33; *P* = .009) underscores the clinically meaningful impact of WBV on overall physical performance. These results indicate that WBV holds potential as an accessible and safe intervention to improve the physical function of older adults, thereby enhancing their quality of life and functional independence.

We recently discussed the efficacy of WBV exercise in people who were bed bound and intensive care unit bound as a countermeasure to prevent physical deconditioning, especially in situations where the ability of patients to cooperate and to exercise is limited.^45^ This is also the case for geriatric patients hospitalized because of an acute exacerbation of chronic obstructive pulmonary disease^46^ and, as reported here and elsewhere, in older people who resided in an institution.^25^ Functional decline experienced in this group of nursing home residents can affect their ability to perform activities of daily living. Recent studies reported that the chair stand ability, a fundamental activity of daily living, is considerably reduced (ie, ~50%) in people older than 70 years.^47^ The CST was recently reported to reflect physical function in older individuals.^48^ Consequently, the significant effects in CST achieved after WBV intervention may have clinical importance (SMD = 0.57), with some studies even showing high effect sizes.^32^ Together with the CST, the European Working Group on Sarcopenia in Older People (EWGSOP) also recommend the use of the TUG Test for the assessment of muscle strength and physical function (ie, mobility) in older adults.^6^ A significant effect was found for the TUG Test in the current study; this finding concurs with those of previous systematic reviews of WBV in older people who resided in an institution^32^ and those who did not reside in an institution.^49^ These improvements were attributed to a greater activation of motor units and a stimulation of the primary endings of the muscle spindle receptors that might cause reflexive contraction,^50^ thereby improving neuromuscular function.

The positive effects of WBV on balance shown in our meta-analysis contrasts with the findings observed by Alvarez-Barbosa et al^25^ for older people who did not reside in an institution. One potential reason for this difference is that the lower initial level of physical performance expected in very old nursing home residents (as in our study) could be more amenable to greater benefits for improving balance after WBV.^22^ Our results of the positive effect of WBV on balance are consistent with the meta-analysis by Rogan et al,^22^ who found that WBV can positively improve static balance in older people who were more mobile and had the potential to improve dynamic balance in older people who were functionally less able.

In our meta-analysis both balance (SMD = 0.41) and the gait score using the Tinetti Test (SMD = 0.26) were significantly improved in the intervention group, although no changes were observed in gait speed. A previous systematic review and meta-analysis reported that WBV was effective in increasing postural control in older people and that these changes could be extended to dynamic motor tasks such as walking.^22^ The lack of effect on gait speed could also be attributed to the heterogeneous methodologies, especially regarding the vibration type, and the differences in the protocols used.

We found lack of consistency and consensus in WBV protocols. Some differences were seen in the WBV training protocols. Interventions varied between 4 weeks and 6 months, usually 2 or 3 sessions per week, with a training session usually consisting of 2 to 10 bouts of 30 to 60 seconds of WBV, with 60 seconds of rest between the bouts. Overall, it is interesting to note that most of the significant changes in strength^19^^,^^37^^,^^41^^,^^42^ or mobility^32^^,^^34^^,^^36^ were achieved with frequencies of 30 to 40 Hz, with only a couple of exceptions^38^^,^^40^; in all cases, a vertical vibration platform, rather than a side-alternating one, was used. However, although the amplitudes were mostly between 2 and 4 mm for improving lower limb muscle strength, in order to enhance mobility, amplitudes were slightly lower (0.9–2 mm). These findings could inform researchers and practitioners in WBV when designing protocols. Programs with moderate frequencies (~25 Hz) and low amplitudes (1–2 mm) progressing to higher frequencies (~30–35 Hz) and higher amplitudes (2–4 mm) should be considered.

An important consideration regarding WBV is whether this intervention is feasible and tolerated by older people, particularly those who are frail and reside in nursing homes. WBV was well tolerated in older fallers with a mean age of 80 years.^51^ In our current systematic review of nursing home residents, most of the included studies reported no notable adverse effects; however, minor tingling or transient itching of the lower limbs,^24^^,^^35^ knee or back pain,^24^^,^^36^ or lower limb soreness^24^^,^^37^ were reported in some studies, suggesting the need to progress more slowly in this vulnerable population. This is consistent with previous reports showing that WBV is safe and well tolerated by older people who do not reside in an institution and can be undertaken independently by many people in care homes.^25^

Some limitations of our systematic review and meta-analysis should be acknowledged. First, some studies could not be included because of a lack of complete data, despite requests to the authors. Second, there were notable variations between effect sizes across the selected studies, raising questions about the importance of controlling for dissimilar methodologies when attempting to aggregate data for the meta-analysis. Further, the study population, although older than 80 years in all cases, were dissimilar in terms of concomitant conditions. Moreover, the amount of heterogeneity in the outcomes related to mobility and balance pooling made the study results challenging and require cautious interpretation. Despite these limitations, our results should be helpful in developing WBV exercise programs for nursing home residents and in designing further research studies in this sector.

## Conclusions

The results of this systematic review and meta-analysis suggest that WBV can be an effective treatment intervention to improve strength, balance, mobility, walking ability, and physical performance of nursing home residents older than 80 years. Further research is needed to define standardized protocols targeting different outcomes in this population group. WBV appears to be a safe, easily performed, and acceptable alternative exercise intervention compared to other exercise regimens in this group of older residents of care homes who are unable to undertake more conventional evidence-based exercises.

## Supplementary Material

2023-0469_R1_Supplementary_Materials_cjt_pzae025

## Systematic Review Registration

This study was registered in the International Prospective Register of Systematic Reviews (PROSPERO) (CRD42021279735).
