# Supplementary material for: Whole-Body Vibration to Improve Physical Function Parameters in Nursing Home Residents Older Than 80 Years: A Systematic Review With Meta-Analysis
Source: Phys Ther. 2024 Feb 29;104(5):pzae025. doi: 10.1093/ptj/pzae025 (PMC11116829; doi:10.1093/ptj/pzae025)
Supplement: 2023-0469_R1_Supplementary_Materials_cjt_pzae025 [file 2023-0469_r1_supplementary_materials_cjt_pzae025.pdf]

Supplementary Materials

Supplementary Table 1. Description of the included studies

Author (year)	Country	Participants enrolled (analized)	Age (mean, sd)	Outcomes	Adherence	Adverse effects
Álvarez-Barbosa 2014	Spain	EG, n=15(11)<br>GC, n=14(11)	EG: 84 ± 3<br>GC: 85 ± 7	TUG; 30-s CST, balance, QoL (Euro-QoL)	73% (11 out of 15) completed 80% sessions	No notable side effects were observed
Beaudart 2013	Belgium	EG, n=31(30)<br>GC, n=31(25)	EG: 82 ± 9<br>GC: 84 ± 7	TUG, balance, gait kinematics, falls.	N=6 (19.4%) dropped out	N=4 (2 hip pain, 1 fracture - malleolus-; n=1 hip replacement unrelated to the study)
Bogaerts 2011*	Belgium	EG, n=29(25)<br>GC, n=28(26)	EG: 80 ± 5<br>GC: 80 ± 5	TUG, balance, 10-m walk test, Shuttle Walk, knee extension strength, Fall risk	>90%, n=16 (72.7%)	N=4 for medical reasons
Bruyere 2005	Belgium	EG, n=22(16)<br>GC, n=20(20)	EG: 84 ± 6<br>GC: 80 ± 7	TUG, balance, QoL (SF-36)	N=6 (27.3%) dropped out	N=2 transient minor tingling of the lower limbs
Buckinx 2014	Belgium	EG, n=31(19)<br>GC, n=31(17)	EG: 82 ± 9<br>GC: 84 ± 7	TUG, balance, gait kinematics, falls.	74% (23 out of 31) completed 91% sessions	N=2 hospitalization; n=2 back pain and sick; n=3 inability
De Bruin 2020	Switzerland	EG, n=9(6)<br>CG, n=8(7)	EG: 86 ± 6<br>GC: 90 ± 6	SPPB (STS, walking speed, balance), Falls (FES-I)	Adherence rate 76.5%. Compliance 100%.	There were no adverse events
Grubbs 2020	US	EG, n=14(10)<br>GC, n=11(10)	EG: 83 ± 6<br>GC: 81 ± 4	TUG, SPPB (STS, walking speed, balance), Isometric handgrip and leg strength, Knee extension force	100% (n = 10 in EG completed the 24 training sessions)	Itchy nose, headache, and lower limb soreness were common during the beginning of training.
Kessler 2014	Switzerland	EG, n=14(13)<br>CG, n=13(11)	EG: 91 ± 7<br>GC: 84 ± 9	SPPB (STS, walking speed, balance), knee flexion	Adherence rate of 90%	No notable side effects were observed

				and extension strength,		
Lam 2018	Hong Kong	EGex, n=25(21)<br>EX, n=24(19)<br>GC, n=24(22)	EGex: 84 ± 7<br>EX: 82 ± 7<br>GC: 80 ± 7	TUG, STS, Balance, 6MWT, proprioception, knee extension strength, and reaction time	77.1% (n=4 in EEGx had an attendance rate <50%)	N=2 hospitalization, n=1, Due to pneumonia<br>No notable side effects were observed
Ochi 2015*	Japan	EG, n=10(10)<br>EQ, n=10(10)	EG: 81 ± 3<br>EQ: 80 ± 3	TUG, 10-m walking, knee extension isometric strength	Attendance rate of 99%	No notable side effects were observed
Sievänen 2014	Finland	EG, n=8(7)<br>CG, n=7(6)	EG: 84 ± 6<br>GC: 84 ± 9	TUG, SPPB (STS, walking speed, balance), handgrip strength	87 % (15 out of 20) measured after the 10-week. Attended >73 %	No notable side effects were observed<br>n=2 died
Sitjà-Rabert 2015	Spain	EG, n=81(67)<br>CG, n=78(64)	EG: 82 ± 8<br>GC: 82 ± 7	Balance, 5STS, TUG, Vmax, falls	Not reported	Pain (18%): knees and lumbar spine. Soreness (13%): legs. Transient itching (0.6%), erythema (1.2%), and edema of the legs (0.6%)
Smith 2016*	US	EG, n=13<br>CG, n=14	EG: 82 ± 5<br>GC: 82 ± 6	Balance, Functional Independence Measure, dynamic muscle strength	Compliance >90%; drop out/attrition due to no reason or lack of time (N = 2)	n=1 death
Verschueren<br>2011	Belgium	EG, n=29(28)<br>CG, n=28(28)	EG: 80 ± 5<br>GC: 80 ± 5	BMD, Isometric and dynamic muscle strength	adherence rate >90%. N=5: <compliance rate (reasons not related to the study)	No notable side effects were observed<br>N= 4 (intolerance confusion, and n=2 died)

CG: control group; EG: Experimental group; FES-I: Falls Efficacy Scale–International; QoL: Quality of life; SPPB: Short Physical Performance Battery; STS: sit and stand test; TUG: Timed up and go.

Supplementary Table 2. Intervention parameters in the included studies

Author	Frequency	Amplitude	Peak acceleration	Vibration type/device	Sessions/wk	Position/exercises	Protocol	Duration	Footwear
Álvarez-Barbosa 2014	30-35 Hz	4		Vertical (YV20RS 700, BH, Spain)	3	Step up and down, lunge, squat, calf raises, left and right pivot in afront and lateral positions	6-12 reps (12-17 min), 45 s rest	8 wks	Not stated
Beaudart 2013	30 Hz	2		Vertical (Vibrosphere)	3	Squat	5 x 15 s,		Shoeless
Bogaerts 2011	30-40 Hz	2	1.6--2.2	Vertical (Powerplate)	3	Squat, deep squat, wide stance squat, toes stand and one legged squat.	2-5 x 15-60 s, 5-60 s rest	24 wks	Not stated
Bruyere 2005	10-26 Hz	3--7		Side-alterning (Galileo 900)	3	Squat + standard exercise program (gait and balance, strengthening of the lower limbs	4 x 60 s, 60 s rest	6 Wks	Not stated
Buckinx 2014	30 Hz	2		Vertical (Vibrosphere)	3	Squat	5 x 15 s, 60 s rest	24 wks	Shoeless
De Bruin 2020	1-12 Hz	3		Side-alterning (Zeptor med plus Noise)	3	Partial squat or stand parallel holding onto the bars	5x 60s, 60 s rest	8 wks	No shoes
Grubbs 2020	25-40 Hz	1		Vertical (Powerplate)	2	partial squats, narrow squats, wide squats, and calf raises.	3 x 10 (50 s), 110 s rest	12 wks	Usual shoes or socks
Kessler 2014	3-6 Hz	3		Side-alterning (Zeptor med plus Noise)	3	Squat or stand parallel holding onto the bars	4 x 60 s, 60	4 wks	Shoeless

							s rest		
Lam 2018	30-40 Hz	0.9	3.4-4.7	Vertical (Fitvibe 600)	3	dynamic semi-squats; heel raise; and single-leg standing, on both lower limbs.	1-4 x 60 s, 60-120 s rest	8 wks	Not stated
Ochi 2015	10-21 Hz	3--7	1.2-12.4	Side-alterning (Galileo 2000)	3	half squat, heel rise, toe up and weight shift training	180 s	12 Wks	Not stated
Sievänen 2014	12-18 Hz	2--8		Side-alterning (Galileo 900)	2	slight squatting; toe raises; lateral weight transfers	1-5 x 60 s, 60 s rest	10 wks	Not stated
Sitjà-Rabert 2015	30-35 Hz	2--4		Vertical (Powerplate)	3	Squat, Squat with semi-tandem position, calf raises, lateral weight transfers, tiptoe-calf raise	6-8 x 30-60 s, 60 s rest	6 wks	Not stated
Smith 2016	30 Hz	1--2		Vertical (Powerplate)	2	Semi-squatting position	3 x 60 s, 60 s rest	12 wks	Not stated
Verschueren 2011	30-40 Hz	2	1.6--2.2	Vertical (Powerplate)	3	Squats (deep, wide-stance, toe stance, and one-legged).	2-5 x 15-60s, 5-60 s rest	24 wks	Not stated

Supplementary Table 3. Assessment of risk of bias for included studies.

Study	Eligibility	Random	Allocation	Inter group	Blinding	Blinding	Blinding	Adequate	Intention to	Between group	Measure of	Total score

Álvarez-Barbosa 2014	Y	1	1	1	0	0	0	0	1	1	1	6
Beaudart 2013	Y	1	0	1	0	0	1	0	1	1	1	6
Bogaerts 2011*	y	1	1	1	0	0	0	1	1	1	1	7
Bruyere 2005	Y	1	0	1	0	0	0	1	1	1	1	6
Buckinx 2014	Y	1	1	1	0	0	1	1	1	1	1	8
De Bruin 2020	Y	1	1	1	0	0	1	1	0	1	1	7
Grubbs 2020	Y	1	0	1	0	0	0	1	0	1	1	5
Kessler 2014	Y	1	1	1	1	0	0	1	0	1	1	7
Lam 2018	Y	1	0	1	0	0	1	1	1	1	1	7
Ochi 2015*	Y	1	0	1	0	0	0	1	0	1	1	5
Sievänen 2014	Y	1	0	1	0	0	1	1	1	1	1	7
Sitjà-Rabert 2015	Y	1	0	1	0	0	1	0	1	1	1	6
Smith 2016*	Y	1	0	1	0	0	0	1	0	1	1	5
Verschueren 2011	Y	1	0	1	0	0	1	1	1	1	1	7

Supplementary Figure. Flow diagram of study selection process according to PRISMA.[26]

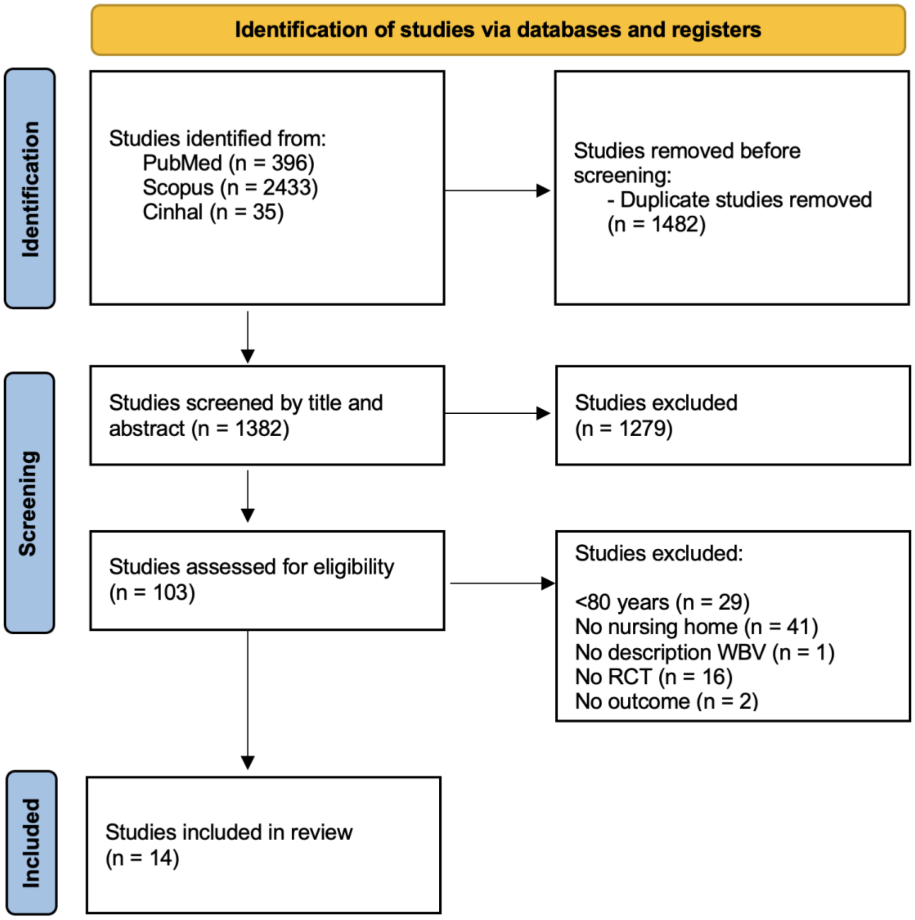

PRISMA 2020 Checklist

Section and Topic	Item #	Checklist item	Location where item is reported
**TITLE**			
Title	1	Identify the report as a systematic review.	1
**ABSTRACT**			
Abstract	2	See the PRISMA 2020 for Abstracts checklist.	1-2
**INTRODUCTION**			
Rationale	3	Describe the rationale for the review in the context of existing knowledge.	3-4
Objectives	4	Provide an explicit statement of the objective(s) or question(s) the review addresses.	5
**METHODS**			
Eligibility criteria	5	Specify the inclusion and exclusion criteria for the review and how studies were grouped for the syntheses.	6
Information sources	6	Specify all databases, registers, websites, organisations, reference lists and other sources searched or consulted to identify studies. Specify the date when each source was last searched or consulted.	5-6
Search strategy	7	Present the full search strategies for all databases, registers and websites, including any filters and limits used.	Annex
Selection process	8	Specify the methods used to decide whether a study met the inclusion criteria of the review, including how many reviewers screened each record and each report retrieved, whether they worked independently, and if applicable, details of automation tools used in the process.	6-7
Data collection process	9	Specify the methods used to collect data from reports, including how many reviewers collected data from each report, whether they worked independently, any processes for obtaining or confirming data from study investigators, and if applicable, details of automation tools used in the process.	7
Data items	10a	List and define all outcomes for which data were sought. Specify whether all results that were compatible with each outcome domain in each study were sought (e.g. for all measures, time points, analyses), and if not, the methods used to decide which results to collect.	6-7
	10b	List and define all other variables for which data were sought (e.g. participant and intervention characteristics, funding sources). Describe any assumptions made about any missing or unclear information.	6-7
Study risk of bias assessment	11	Specify the methods used to assess risk of bias in the included studies, including details of the tool(s) used, how many reviewers assessed each study and whether they worked independently, and if applicable, details of automation tools used in the process.	7
Effect measures	12	Specify for each outcome the effect measure(s) (e.g. risk ratio, mean difference) used in the synthesis or presentation of results.	7
Synthesis methods	13a	Describe the processes used to decide which studies were eligible for each synthesis (e.g. tabulating the study intervention characteristics and comparing against the planned groups for each synthesis (item #5)).	7
	13b	Describe any methods required to prepare the data for presentation or synthesis, such as handling of missing summary statistics, or data conversions.	8
	13c	Describe any methods used to tabulate or visually display results of individual studies and syntheses.	7
	13d	Describe any methods used to synthesize results and provide a rationale for the choice(s). If meta-analysis was performed, describe the model(s), method(s) to identify the presence and extent of statistical heterogeneity, and software package(s) used.	9
	13e	Describe any methods used to explore possible causes of heterogeneity among study results (e.g. subgroup analysis, meta-regression).	9
	13f	Describe any sensitivity analyses conducted to assess robustness of the synthesized results.	9
Reporting bias assessment	14	Describe any methods used to assess risk of bias due to missing results in a synthesis (arising from reporting biases).	Item 11
Certainty assessment	15	Describe any methods used to assess certainty (or confidence) in the body of evidence for an outcome.	11-13

PRISMA 2020 Checklist

Section and Topic	Item #	Checklist item	Location where item is reported
**RESULTS**			
Study selection	16a	Describe the results of the search and selection process, from the number of records identified in the search to the number of studies included in the review, ideally using a flow diagram.	8
	16b	Cite studies that might appear to meet the inclusion criteria, but which were excluded, and explain why they were excluded.	Fig. 1
Study characteristics	17	Cite each included study and present its characteristics.	Table 1-2
Risk of bias in studies	18	Present assessments of risk of bias for each included study.	10
Results of individual studies	19	For all outcomes, present, for each study: (a) summary statistics for each group (where appropriate) and (b) an effect estimate and its precision (e.g. confidence/credible interval), ideally using structured tables or plots.	Table 1-2
Results of syntheses	20a	For each synthesis, briefly summarise the characteristics and risk of bias among contributing studies.	10
	20b	Present results of all statistical syntheses conducted. If meta-analysis was done, present for each the summary estimate and its precision (e.g. confidence/credible interval) and measures of statistical heterogeneity. If comparing groups, describe the direction of the effect.	10
	20c	Present results of all investigations of possible causes of heterogeneity among study results.	11-13
	20d	Present results of all sensitivity analyses conducted to assess the robustness of the synthesized results.	11-13
Reporting biases	21	Present assessments of risk of bias due to missing results (arising from reporting biases) for each synthesis assessed.	11-13
Certainty of evidence	22	Present assessments of certainty (or confidence) in the body of evidence for each outcome assessed.	Fig. 2-7
**DISCUSSION**			
Discussion	23a	Provide a general interpretation of the results in the context of other evidence.	13
	23b	Discuss any limitations of the evidence included in the review.	13
	23c	Discuss any limitations of the review processes used.	15
	23d	Discuss implications of the results for practice, policy, and future research.	15
**OTHER INFORMATION**			
Registration and protocol	24a	Provide registration information for the review, including register name and registration number, or state that the review was not registered.	5
	24b	Indicate where the review protocol can be accessed, or state that a protocol was not prepared.	5
	24c	Describe and explain any amendments to information provided at registration or in the protocol.	Pending
Support	25	Describe sources of financial or non-financial support for the review, and the role of the funders or sponsors in the review.	1
Competing interests	26	Declare any competing interests of review authors.	1
Availability of data, code and other materials	27	Report which of the following are publicly available and where they can be found: template data collection forms; data extracted from included studies; data used for all analyses; analytic code; any other materials used in the review.	On request

From: Page MJ, McKenzie JE, Bossuyt PM, Boutron I, Hoffmann TC, Mulrow CD, et al. The PRISMA 2020 statement: an updated guideline for reporting systematic reviews. BMJ 2021;372:n71. doi: 10.1136/bmj.n71

For more information, visit: http://www.prisma-statement.org/
